# Overexpression of milk thistle *SOD* gene enhances drought tolerance in tobacco by improving photosynthesis and photoprotection

**DOI:** 10.1038/s41598-025-31510-3

**Published:** 2025-12-11

**Authors:** Rahele Ghanbari Moheb Seraj, Masoud Tohidfar, Keyvan Esmaeilzadeh-Salestani, Sasan Aliniaeifard, Asadollah Ahmadikhah, Mahdi Behnamian, Zahra Khazaei, Mehrdad Shahbazi, Morteza Parvandi, Ehsan Sohrabi

**Affiliations:** 1 Department of Horticultural Sciences, Faculty of Agriculture and Natural Resources, University of Mohaghegh Ardabili, Ardabil, Iran; 2 Department of Cell & Molecular Biology, Faculty of Life Sciences & Biotechnology, Shahid Beheshti University, Tehran, Iran; 3 Institute of Technology, University of Tartu, Nooruse 1, Tartu, 50411 Estonia; 4 Department of Horticulture, College of Agricultural Technology (Aburaihan), University of Tehran, Tehran, Iran; 5 Controlled Environment Agriculture Center, College of Agriculture and Natural Resources, University of Tehran, Tehran, Iran; 6 Institute of Experimental Botany of the Czech Academy of Sciences, Centre of Plant Structural and Functional Genomics, Šlechtitelů 31, Olomouc, 77900 Czech Republic; 7 Department of Agriculture, Medicinal Plants and Drugs Research Institute, Shahid Beheshti University, Tehran, Iran

**Keywords:** Transgene, Stomata, Photosynthesis, Antioxidant enzyme, Biotechnology, Expression systems, Gene delivery

## Abstract

Superoxide dismutase (SOD) is involved in the plant’s primary defense mechanism against the adverse effects of reactive oxygen species (ROS), maintaining ROS homeostasis within cells. Therefore, it is essential to investigate and compare the stress tolerance mechanism in both tolerant and sensitive plants. This study investigates the drought stress tolerance of Tobacco following transferring of a *SOD* gene obtained from a drought-tolerant plant. The *SOD* gene was isolated from the Milk thistle (*Silybum marianum* L.) plant (*SmSOD*), cloned into the pBI121 (Binary Agrobacterium plasmid) expression vector, and subsequently transformed into *Agrobacterium tumefaciens*. Pre-cultivated Tobacco (*Nicotiana tabacum* L.) plants were inoculated with the recombinant bacteria. Following validation of transgene integration via PCR, plants subjected to drought stress corresponding to ~ 50% field capacity for 5 days. Results demonstrated that the expression of *SOD* and its enzyme activity in drought-imposed transgenic plants were respectively 5 and 1.8 times higher than its expression and activity in wild-type plants. Imaging of chlorophyll fluorescence showed that drought-imposed transgenic plants had a higher maximum quantum yield of photosystem II (F_v_/F_m_) and Non-photochemical quenching (NPQ) compared with wild-type plants. Stomatal density in drought-imposed plants was lower than in control plants, consistent across both transgenic and wild-type groups. Stomatal width decreased under stress in both plant types, with transgenic plants showing smaller width than wild-type plants. Stomatal length showed no significant differences between transgenic and wild-type plants or between drought-stressed and control conditions. In conclusion, *SmSOD* gene transfer from the Milk thistle to Tobacco plants increased the drought tolerance of the transgenic plants; this shed light on the path toward reaching drought-tolerant crops. These findings highlight the potential of SmSOD as a key gene for improving drought resistance in crops, offering promising applications in developing drought-tolerant crops for agriculture in water-scarce regions.

**Supplementary Information:**

The online version contains supplementary material available at 10.1038/s41598-025-31510-3.

## Introduction

Water is a critical component in ensuring the viability and sustainability of life across various living organisms. In plants, drought can be characterized as a physiological condition resulting from inadequate soil hydration, leading to a deficiency in water availability for photosynthetic organisms, which adversely influences their biological processes^1–3^. Drought stress causes the formation of reactive oxygen species (ROS), consisting of free radicals, including hydroxyl and superoxide radicals, or non-radicals such as singlet oxygen and hydrogen peroxide^4^. The ROS production can result in oxidative damage to essential molecules such as proteins, membrane lipids, and DNA, potentially disrupting normal plant cell functions^5^. When plants face oxidative stress, they initiate different molecular, biochemical, physiological, and morphological changes mediated by ROS signaling^6,7^.

Numerous studies have been dedicated to investigating both ROS generation and collecting mechanisms in plants, alongside the roles of these mechanisms throughout plant development and responding to either abiotic or biotic stresses^8,9^. To mitigate oxidative damage within plant cells, the presence of protective enzymes, including catalase (CAT), superoxide dismutase (SOD), and peroxidase (POD), is fundamental in maintaining ROS homeostasis^10^. Currently, investigations have predominantly concentrated on the function of SOD as the initial enzyme involved in the enzymatic antioxidant defense pathway^11^.

The SODs, as widespread metalloenzymes, facilitate the conversion of superoxide radicals into hydrogen peroxide (H_2_O_2_) and molecular oxygen (O_2_). Given the potential of superoxide radicals to evolve into highly reactive hydroxyl radicals, SODs significantly contribute to plants defense mechanisms against oxidative stress^12,13^. These enzymes are classified into three categories based on the metal cofactors they contain: iron SODs (FeSOD), copper/zinc SODs (Cu/ZnSOD), and manganese SODs (MnSOD)^14^. Inside plant cells, different isoforms of these enzymes, including FeSOD, MnSOD, and multiple variants of Cu/ZnSOD, are nuclear-encoded and distributed across various subcellular compartments, each distinguished by its specific metal cofactor^15^. It has shown that their functions are key components of the plant’s abiotic stress tolerance machinery. The Cu/ZnSOD has attracted more investigative focus regarding its function in abiotic stress tolerance than the MnSOD^16^. Among the different isoforms of superoxide dismutase, Cu/ZnSOD plays a broader role in regulating oxidative balance in plants due to its diverse localization in the cytosol, chloroplasts, and peroxisomes. While MnSOD is mainly active in mitochondria and FeSOD is found primarily in chloroplasts, Cu/ZnSOD occurs in multiple organelles and serves as the first line of defense against superoxide accumulation in the cell^17^. Moreover, Cu/ZnSOD genes usually exhibit higher sensitivity to abiotic stresses such as salinity, drought, and temperature, and their expression often increases during the early stages of stress response. Several studies have shown that overexpression of Cu/ZnSOD genes in different plant species enhances stress tolerance and reduces oxidative damage^18^.

**Fig. 1:**
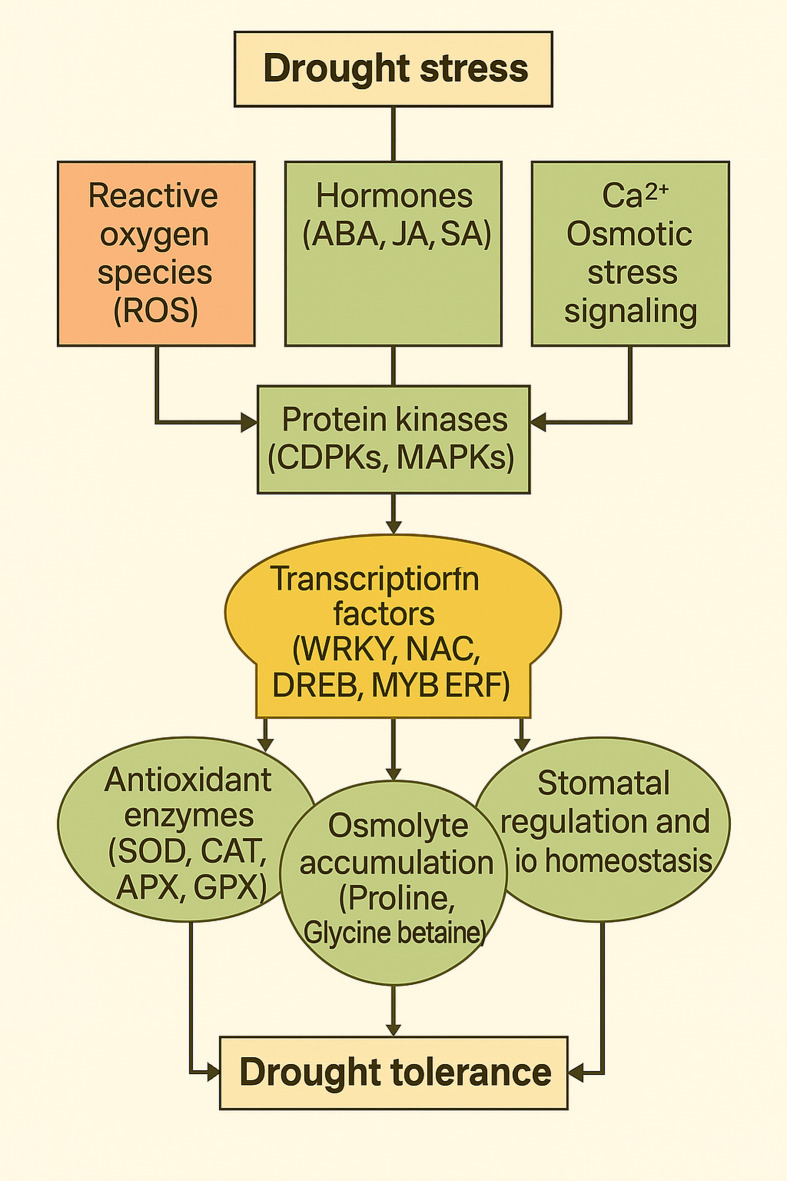
Schematic representation of plant signaling networks and physiological responses to drought stress. Drought stress activates reactive oxygen species (ROS), hormonal signaling (ABA, JA, SA), calcium, and osmotic stress signaling pathways, which lead to the activation of protein kinases (CDPKs, MAPKs). These pathways regulate transcription factors (WRKY, NAC, DREB, MYB ERF), which in turn modulate the activities of antioxidant enzymes (SOD, CAT, APX, GPX), promote osmolyte accumulation (proline, glycine betaine), and control stomatal regulation and ion homeostasis, thereby collectively enhancing drought tolerance and maintaining plant homeostasis.

Drought conditions stimulate activating genes engaged in stress, fostering the accumulation of metabolically functional compounds and initiating the biosynthesis of particular proteins^19^. Plants employ intricate mechanisms to alleviate the detrimental effects associated with drought conditions^20^. A large number of genes that responded to drought stress have been found, cloned, and subsequently applied as potential candidates in the field of genetic engineering^21^. For instance, genes encoding proteins critical for abiotic stress resilience include antioxidative enzymes (SOD, CAT, APX, GPX, DHAR and GR), enzymes necessary for osmolyte biosynthesis (Protein kinases, Amino acid synthases, Glucose-6-phosphate dehydrogenase, Sorbitol dehydrogenase and Phenylalanine ammonia-lyase), and enzymes that play a role in regulating and transcribing genes responsible for stress (RNA polymerase, Histone acetyltransferases (HATs), Histone deacetylases (HDACs), DNA methyltransferases, Protein kinases (e.g., MAPK), Chromatin remodeling enzyme and Transcription factors (e.g., WRKY, CBF, MYB, bHLH, ERF)^22–26^. The regulatory pathway for drought stress tolerance in plants is illustrated in Fig. 1.

The initial physiological adaptation of plants under drought stress primarily involves stomatal closure to reduce transpiration, serving as a crucial strategy to minimize water loss^27,28^. However, this response concurrently limits CO_2_ uptake, thereby decreasing photosynthetic efficiency^29,30^. Reduced transpiration further restricts nutrient uptake and translocation from roots to aerial tissues, leading to ionic imbalance within plant cells^31,32^. These disruptions adversely affect various physiological processes, particularly photosynthesis, which is directly associated with plant growth and productivity^33,34^. Moreover, drought stress impairs chloroplast integrity and chlorophyll biosynthesis, resulting in decreased photosynthetic capacity and the onset of oxidative stress within plant tissues^34,35^.

Enhancing drought tolerance through transgenic approaches has emerged as an effective strategy to improve plant resilience. Overexpression of SOD genes in transgenic plants has been shown to increase enzymatic activity, promote ROS scavenging, and mitigate oxidative stress, particularly in the context of ROS signaling within chloroplasts^36^. Milk thistle is recognized as a drought-tolerant species, and its unique characteristics in withstanding environmental stress make it a promising donor species for genetic engineering to enhance drought tolerance in other plants.

In this study, we selected the *SmSOD* gene from *Silybum marianum* due to its high antioxidant activity and reported role in drought tolerance in its native plant. Tobacco was chosen as the host species because of its economic importance and sensitivity to drought, making it a suitable model for studying stress responses.

The aim of this study was to investigate the effect of *SmSOD* overexpression on drought tolerance in Tobacco. We systematically assessed physiological parameters (photosynthetic efficiency and stomatal morphology), enzymatic activity (SOD activity), and transcriptional level (SmSOD expression) under both control and drought stress conditions to provide a comprehensive understanding of *SmSOD*-mediated stress adaptation.

## Materials and methods

### Experimental design and sampling

The experiment was performed at the experimental field of Shahid Beheshti University, Tehran, Iran (51.23° N and 35.48° E). The soil of the experimental site was classified as loam with a pH of 7.27 and an electrical conductivity (EC) of 2.13 dS/m. The soil contained 29.5% clay, 35% silt, and 44% sand, with a field capacity of 39% and available water content of 33%. The experimental design and related details have been previously described comprehensively^37^. To isolate the target *SmSOD* gene, Milk thistle plants cultivated under field conditions while under severe drought stress (40% field capacity (FC)) were used. Sampling began eight days after the application of the stress condition. For this purpose, three plants were randomly selected, and medium-sized leaves in the middle parts of the stem were collected. The leaves were placed in liquid nitrogen, and thereafter kept at − 80 °C until further analysis.

### Primer design and isolation of *SmSOD* gene from milk Thistle

Since the *SmSOD* gene was not identified in Milk thistle and its sequence was unavailable on the NCBI website, the sequence of this gene was identified in plants from the same family as Milk thistle, such as Sunflower and Safflower. Subsequently, these sequences were subjected to BLAST^38^ on the NCBI site to identify similar sequences. The obtained gene sequences were then stored and aligned using the T-COFFEE Multiple Sequence Alignment Server^39^. To design primers, conserved regions were used at the start and end of the gene sequence, encompassing the start and stop codons. Due to the unknown sequence of this gene in Milk thistle, the primers were designed degenerately from the total obtained sequences.

Vector NTI^40^ and Oligo7^41^ software were employed for the evaluating parameters such as melting temperatures (TM), GC content, stem-loop structures, homodimer, and heterodimer of primers, which were subsequently synthesized by Bioneer Company (South Korea). The details of the primers for *SmSOD* and housekeeping genes, including sequences and other characteristics, are presented in Table 1. To extract RNA, 0.2 g of fine powdered fresh leaves (using liquid nitrogen) were used employing the RNX Plus kit (RB429A, Sinaclon, Iran). Then, RNA samples were treated with DNase I (RB125A, RNA, Iran) to remove any potential genomic contamination. The synthesis of cDNA was carried out in a 20 µL reaction volume using reagents from a cDNA synthesis kit (RB225A, Parstous, Iran), following the provided manufacturer’s protocol, and synthesized cDNA was subsequently kept at − 20 °C until further analysis.

The PCR was conducted on the target gene and housekeeping genes using specific primers, as listed in Table 1. PCR mixtures included 10 µL of Master Mix (RNA, Iran), 1 µL of each forward and reverse primer,1 µL of cDNA, and 7 µL water. The thermal cycler was programmed for an initial denaturation phase at 94 °C for 5 min, succeeded by 35 cycles at 94 °C for 20 s, 57 °C for 15 s, and 72 °C for 20 s, with a final extension period for 5 min at 72 °C. After the amplification, the PCR products were assessed through electrophoresis on a 1% agarose gel. After electrophoresis, the DNA fragments were carefully cut out from the gel using a sterile scalpel and placed into a 1.5 mL microtube. DNA purification was done using the AccuPrep^®^ Gel Purification Kit (Bioneer Pacific, K-3038, Germany), adhering to the manufacturer’s protocol. Successful purification was verified through a subsequent round of PCR on the purified solution.

**Table 1:** Characteristics of primers used in this experiment.

Primer Name	Primer sequence	Primer length	TM
*SmSOD*^a^-F	AATGGTGAAGGCTGTTG	17	42.7
*SmSOD*^a^-R	ATGATCAACCCTGCAATCC	19	49.3
*SmSOD*^b^-F	AGTTCGCCATGCTGGTGATC	20	54.8
*SmSOD*^b^-R	TCCAATGATACCACATGCAAC	21	50.6
*M13*-F	GTTTTCCCAGTCACGAC	17	41.7
*M13*-R	CAGGAAACAGCTATGAC	17	36.2
*18SrRNA*-F	ATGATAACTCGACGGATCGC	20	56.7
*18SrRNA*-R	CTTGGATGTGGTAGCCGTTT	20	57.2
*NPTII*-F	GAAGAACTCGTCAAGAAGGCGATAGA	26	58.3
*NPTII*-R	ATTGAACAAGATGGATTGCACGCAGGTTCT	30	66.8

^a^ *SmSOD* primers are designed for gene isolation.

^b^ *SmSOD* primers are designed to determine gene expression by qRT-PCR

TM: melting temperature, the temperature at which half of the DNA duplex dissociates into single strands, used to indicate primer binding stability in PCR.

### Construction of Recombinant vector (pTG19- *SmSOD*)

The purified product was inserted into the pTG19 plasmid vector (Sinaclon, PR911643, Iran) according to the manufacturer’s guidelines. Subsequently, the plasmid, once ligated, transformed into a competent *Escherichia coli* (*E. coli*) DH5α strain using the thermal shock method^42^. This was followed by incubation in Luria-Bertani (LB) broth medium without antibiotics for one hour at 37 °C. Blue-white screening, PCR, enzymatic digestion, and sequencing were employed to verify the gene’s successful cloning. Subsequently, the plasmid was isolated from the bacteria constituting the white colonies using the GTP Plasmid DNA Extraction Kit (Gene Transfer Pioneers, IRAN), followed by digestion with the BamHI enzyme. These reactions were incubated at 37 °C for 16 h. The products obtained were examined through electrophoresis (1% agarose gel), and their sizes were compared against a 1 kb DNA ladder (SL7052, Sinaclon, IRAN).

### Sequencing, assembling, and gene submission to the NCBI website

The plasmid pTG19, containing the *SmSOD* gene, was sent to Bioneer company (South Korea) for Sanger dideoxy sequencing in both directions ($5^{\prime}\to{3}^{{\prime}}$ and $3^{\prime}\to{5}^{{\prime}}$) with *M13* primers (forward and reverse). The length of reads was 1000 bp in each direction. The forward and reverse sequences obtained from sequencing were assembled by Vector NTI Advance^®^ 11.0 and ChromasPro 2.0 software^43^. Then, the resulting sequence was subjected to BLAST at the NCBI to confirm the accuracy of the sequence. To submit the *SmSOD* gene sequence to the NCBI database, the coding sequences (CDS) of selected sequences derived from Milk thistle were first extracted by Vector NTI software, and then the resulting sequence was submitted to NCBI following the site’s submission guidelines.

### Construction of expression Recombinant vector (pBI121 + *SmSOD*)

In this study, the plasmids pTG19 + *SmSOD* (recombinant) and pBI121, serving as the expression vector, were subjected to enzymatic digestion using BamHI enzymes. This procedure was succeeded by the purification of the digested products—the pBI121 plasmid and the *SmSOD* fragment—employing the AccuPrep^®^ Gel Purification Kit. The *SmSOD* fragment underwent ligation into the BamHI-digested pBI121 plasmid, according to Sambrook and Russell’s^44^ method. Then, the ligated product was introduced into *E. coli* DH5α strain through standard transformation protocols. The culture of the transformed *E. coli* was performed in LB broth medium without antibiotics using a shaker incubator at 37 °C for one hour. The transformed bacteria were spread onto LB agar plates supplemented with kanamycin at 50 mg/L and incubated at 37 °C for 16 h. To verify the incorporation of the recombinant pBI121 plasmid, PCR amplification and restriction digestion using the EcoRI and HindIII enzymes were performed^45,46^.

### Conformation of *Agrobacterium tumefaciens* LBA4404 and tobacco inoculation

The transformation of the recombinant expression plasmid into *A. tumefaciens* was accomplished using the freeze-thaw method described by Höfgen and Willmitzer^47^. Moreover, *A. tumefaciens* strain LBA4404 facilitated the genetic modification of Tobacco (*Nicotiana xanthi*) via an adapted leaf disc method^48^. Following sterilization using a 0.02% HgCl_2_ solution for 12 min, Tobacco seeds were subsequently cultured in Murashige and Skoog (MS) culture medium without any hormones. After a growth period of 10 days, the resulting plantlets were separated and sliced into smaller fragments using a scalpel. These fragments were then prepared for bacterial inoculation procedures^49^.

Three days before the inoculation with *A. tumefaciens*, a pre-culture of leaf disc explants using the MS medium was done. Following the pre-culture period, *A. tumefaciens* (LBA4404 with OD_600_ = 0.7) was used to inoculate these wounded explants for 7 min. The explants were dried using filter paper and transferred to a co-culture medium. This medium was enriched with 0.2 mg/L of Indole-3-acetic acid (IAA) and 2.5 mg/L of 6-Benzylaminopurine (BAP) to facilitate direct somatic embryogenesis, as well as 1 mg/L of BAP and 0.1 mg/L of 1-Naphthaleneacetic acid (NAA) aimed at direct shoot induction for 3 days. Afterward, the explants were placed onto a selection medium that consisted of a full MS supplemented with 1 mg/L of BAP and 0.1 mg/L of NAA for direct shoot induction as well as 50 mg/L of kanamycin and 150 mg/L of cefotaxime. Subculture of the explants was carried out every 10 days. Root induction of the explants was performed using a half MS medium, which included 0.1 mg/L of NAA. The growth chamber maintained at 25 °C with a photoperiod of 16 h of light and 8 h of darkness, was used for all tissue culture experiments. Putative transgenic plants that demonstrated appropriate growth characteristics were used for subsequent experimental analysis^50^. Specific primers were used to perform PCR on the putative transgenic plants. This process started with grinding fresh leaf and shoot tissues obtained from putative transgenic lines using liquid nitrogen. The genomic DNA was extracted using the Dellaporta et al.^51^ protocol. The PCR targeted the *SmSOD*, *18SrRNA*, and *NPTII* genes to ascertain the transgenic status of the lines under investigation. To validate the absence of contamination and establish negative and positive controls, water samples (PCR mixture without DNA template), wild-type plant material, and a recombinant plasmid were used, respectively. A total of 100 leaf disc explants were inoculated, from which 20 transgenic plants were generated.

### Tobacco plant cultivation and drought application

Initially, transgenic and control tobacco plants were grown in a controlled laboratory environment (phytotron). Once the plants reached the four-leaf stage, they were transferred to pots containing perlite and covered with a plastic sheet, which was misted with water daily. To facilitate acclimatization, small valves were made in the plastic cover, which was then gradually removed while maintaining a regular watering schedule.

Subsequently, the plants were transferred to larger pots filled with a mixture of leaf mold, perlite, and sand. At this stage, sparing irrigation was replaced with regular watering, and the plants were gradually transitioned from the phytotron to the laboratory and then to the greenhouse.

After full acclimatization, at the eight-leaf stage, the plants were subjected to drought stress treatments. For this purpose, a total of 20 plants were used, consisting of 10 transgenic plants and 10 control plants. Of each group, 5 plants were subjected to drought stress (irrigated at five-day intervals: ~50% Field Capacity), and 5 plants were watered regularly. Finally, the plants were used for various downstream analyses; three biological replicates per treatment were selected, and sample collection was carried out according to the requirements of each assay.

### Molecular and physiological characteristics

#### Gene expression analysis

In this study, RNA was extracted from 0.2 g of fresh Tobacco leaves using an RNA extraction kit (RB1001, RNA, Iran), followed by DNase I (RB125A, RNA, Iran) treatment to eliminate probable genomic contamination. The quantity and quality of isolated RNA were evaluated using a NanoDrop 1000 spectrophotometer (Thermo Scientific, USA) and electrophoresis in a 1% agarose gel. Extracted RNA served as a template for synthesizing the cDNA, utilizing a cDNA synthesis kit (RB125A, RNA, Iran) according to manufacturer guidelines. Then, the cDNA was stored at -20 °C until further analysis. Primers were designed by considering regions proximal to the polyadenine tail, covering 150 to 250 bp for qPCR. Parameters including the Tm, GC content, stem-loop structures, homodimer, and heterodimer formation were assessed utilizing Oligo software (version 7.60) and Vector NTI^®^ Express Designer Software (version 11). The reference gene was *18SrRNA* (GenBank ID: OR083346). Table 1 lists primer sequences along with additional details are listed.

RT-qPCR amplifications were carried out employing the Rotor-Gene 2000 apparatus (Corbett Life Science, Australia) by utilization of SYBR^®^ Green Real-Time PCR Master Mix (RB120, RNA, Iran). A total volume of 20 µL was used to prepare the reactions, which included 10 µL of the 2x SYBR solution, 1 µL of cDNA, 1 µL of each primer at a concentration of 20 nmol, and 7 µL of RNase-free water. The thermal cycling program began with a 5-min initial denaturation phase at 95 °C, succeeded by 35 amplification cycles (1 min of denaturation at 95 °C, 15 s of annealing at 57 °C, and 20 s of extension at 72 °C). Specificity verification for each amplicon was obtained by evaluating post-amplification melting curves, ranging between 60 and 95 °C. The cycle threshold (Ct) values, as well as primer efficiency, were obtained using the LinRegPCR software (11.0)^52^. Moreover, the calculation for relative expression levels was conducted using the Relative Expression Software Tool (REST) (2009), according to the Pfaffl method^53^, incorporating the efficiency of the primers (E) in the following formula, as described by Livak et al.^54^:

$${\mathrm{Gene}}\;{\mathrm{expression}}\;{\mathrm{ration}} = \left( {{\mathrm{E}}_{{{\mathrm{Gene}}}} } \right)^{{\Delta ct\;{\mathrm{Gene}}}} /\left( {{\mathrm{E}}_{{{\mathrm{Ref}}}} } \right)^{{\Delta ct\;{\mathrm{Ref}}}}$$

Three biological and three technical replicates were used to perform all qRT-PCR analyses to ensure the reliability and reproducibility of data.

#### Chlorophyll fluorescence measurement

Fully developed young leaves were used in this experiment to measure parameters obtained from chlorophyll fluorescence imaging in both normal and stress-induced plants. While still attached to their respective plants, these leaves underwent a 20-min dark adaptation. Following this, their slow chlorophyll fluorescence induction was slowly measured using a FluorCam (FluorCam FC 1000-H, Photon Systems Instruments, PSI, Czech Republic) equipped with a CCD camera and four fixed LED panels, which provided measuring pulses and induced saturating flashes. The maximum quantum yield of photosystem II (F_v_/F_m_) was calculated using a specialized method^55,56^. Chlorophyll fluorescence measurements began during exposure to short flashes in darkness, followed by a saturating pulse (3900 PPFD) at the end, which stopped the electron transport by reducing quinone acceptors^57^. The protocol involved capturing two sets of fluorescence readings: one during exposure to the saturating flash (Fm) and the other averaged throughout short flashes in darkness (F0). The calculation of F_v_/F_m_ was performed employing the following equation:

$${\mathrm{F}}_{{\mathrm{v}}} /{\mathrm{F}}_{{\mathrm{m}}} = \left( {{\mathrm{F}}_{{\mathrm{m}}} - {\mathrm{F}}_{0} } \right)/{\mathrm{F}}_{{\mathrm{m}}}.$$

To quantify non-photochemical quenching (NPQ), the maximum fluorescence in light-adapted steady state $\left({F}_{m}^{\prime}\right)$ Wasw also taken. Using this measurement, the NPQ was determined based on the following equation^58^:

$$\mathrm{NPQ} = ({\text{F }}_{\rm m}/{\text{ F}}_{\rm m}^{\prime} )-1$$

The data was calculated using FluorCam software (version 7) (PSI, Czech Republic).

#### Measurement of stomata morphological parameters

The fully developed young leaves from both the control group and those subjected to drought stress treatments were selected for stomatal length, width, index, diversity, and pore width measurements, according to Aliniaeifard and van Meeteren’s^59^ method. For microscopic analysis, segments of the leaves, positioned equally from the leaf’s edges and located halfway between the apex and the base, were selected. A nail polish was used to cover the lower epidermis of fully developed young leaves. After 10 min, the dried polish was removed using transparent sticky tape. Subsequently, samples of the dried polish adhered to the sticky tape were affixed to microscope slides to examine stomatal morphological characteristics under a light microscope.

Stomatal parameters were quantitatively assessed utilizing the Omax top-view software (version 3.5), followed by a detailed analysis with ImageJ software (U.S. National Institutes of Health, Bethesda, MD) (1.8.0). This evaluation included stomatal width and length, pore width and length, and stomatal density (SD) measurements. Fifty stomata from leaves subjected to each specific treatment were investigated. Stomatal density is defined as the frequency of stomata per unit of leaf surface area^60^. Moreover, the analysis included calculating the stomatal index, a metric denoting the ratio of stomatal pores to the total number of epidermal cells (ED), established as a percentage^61^. The stomatal index was calculated following an equation presented by Smith et al.^62^, incorporating SD and the density of ED cells per unit area of the leaf surface.

$${\mathrm{Stomatal}}\;{\mathrm{index}} (\%) = \left( {{\mathrm{SD}}/{\mathrm{SD}}} + {\mathrm{ED}} \right) \times 100.$$

#### Determination of *SmSOD* enzymatic activity

At first, 1 g of leaves samples was subjected to liquid nitrogen and ground into 10 mL of extraction buffer comprised of 0.1 M phosphate buffer at pH 7.5, supplemented with 0.5 mM EDTA. Next, the homogenate was filtered through a quadruple cheesecloth layer and centrifuged at 15,000×*g* for 20 min. All procedural steps dedicated to the extraction of enzymes were conducted at 4 °C to ensure enzymatic integrity. The assessment of total SOD activity was carried out by measuring the enzyme-mediated suppression of nitro blue tetrazolium (NBT) reduction. This was performed by adding two mM riboflavin (0.1mL) into the reaction mixture (3 mL), which consisted of 0.1 mM EDTA, 13.33 mM methionine, 75 µM NBT, and 50 mM phosphate buffer (pH 7.8), 50 mM sodium carbonate, and 0.1mL of the enzyme extract. Subsequent exposure of the mixture to two 15 W fluorescent lamps for 15 min initiated the reaction. Quantitative absorbance measurement was done at a wavelength of 560 nm, with one unit of enzyme activity defined as the requisite amount of enzyme that obtains a 50% reduction in absorbance compared with control tubes without the enzyme^63^.

### Statistical analysis

All molecular and physiological data were analyzed using R software version 3.5.3^64^. Across all analyses, each treatment included three biological replicates, and each biological replicate was measured in three technical replicates. Data normality was verified using the Shapiro–Wilk test from the *stats* package. ANOVA was performed on the data for a completely randomized design (CRD), with treatments considered as fixed effects and replicates as random effects. Furthermore, Duncan’s multiple range test was used for mean comparisons, utilizing the *agricolae* package, with a significance level of 5%^65^. All figures and tables were generated using Microsoft Word and Excel 2013.

## Results

### Cloning of *SmSOD* gene into pTG19 and pBI121 vector

The *SmSOD* gene fragment was cloned into the pTG19 cloning vector to generate more gene copies and create sticky ends for ligation with digestion sites. After 16 h of incubation at 37 °C, white colonies appeared on the E. coli plates, indicating successful cloning (Fig. S1a). To verify whether the *SmSOD* gene is present in these colonies, a digestion with the BamHI enzyme was conducted. The gel electrophoresis of the digestion product validated that the *SmSOD* gene is present in the pTG19 vector. The pTG19 vector map indicates a sequence length of 2880 bp, which increased to 3342 bp after inserting the *SmSOD* gene. Following digestion with the BamHI enzyme, the fragment lengths were 462 bp and 2880 bp, as confirmed by the gel photo (Fig. S2a). To validate the correctness of the *SmSOD* gene, the vector containing the gene was sequenced, and after assembly, a BLAST search on the resulting gene sequence was performed. BLAST analysis of the obtained sequence demonstrated high similarity with SmSOD genes from related plant species, confirming that the cloned gene encodes a Cu/Zn-superoxide dismutase (Cu/Zn-SOD). Subsequently, the 462 bp sequence of the *SmSOD* gene isolated from Milk thistle was submitted to the NCBI site with the accession number MG893090.

**Fig. 2:**
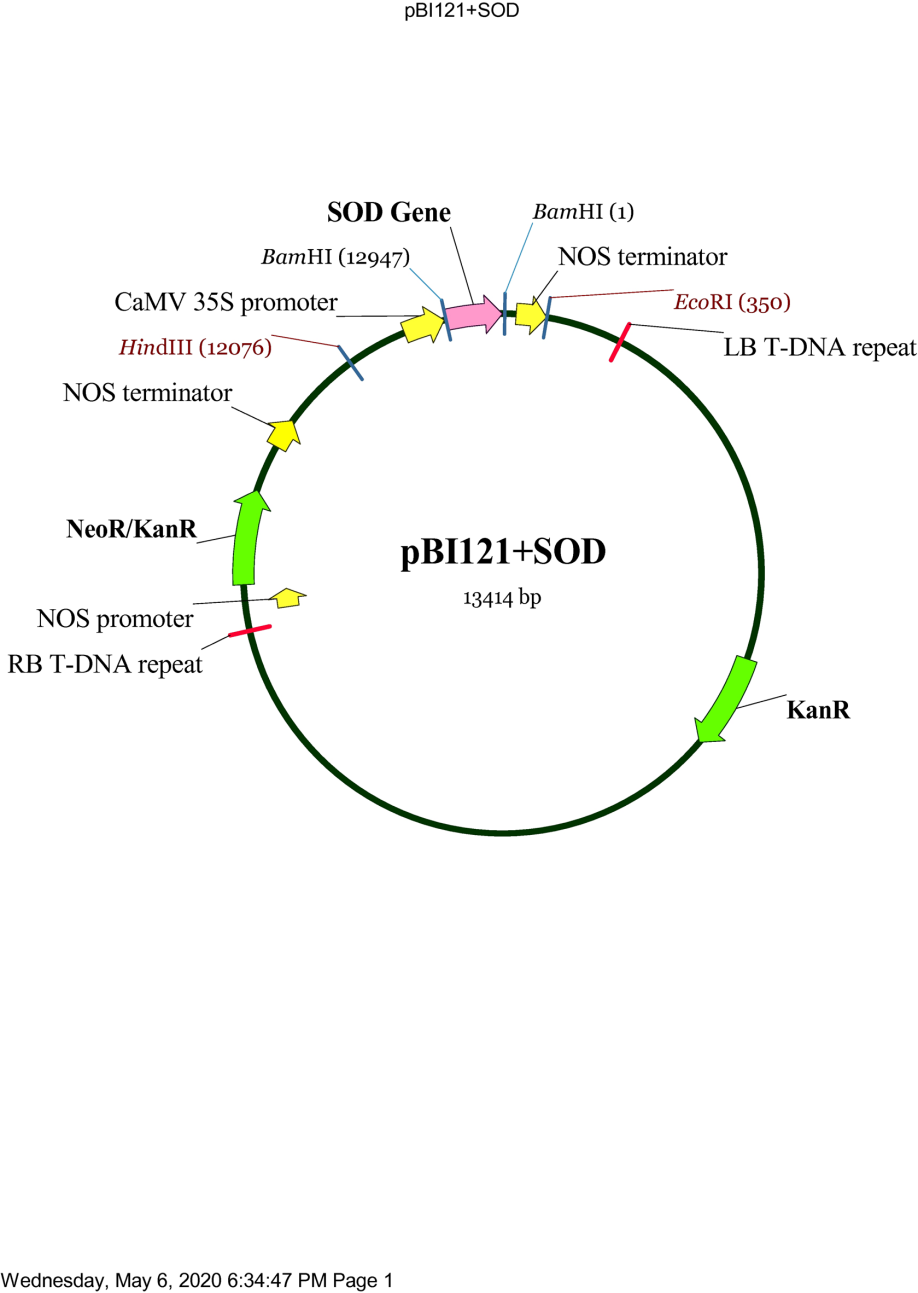
Schematic representation of the pBI121 vector with the *SmSOD* gene inserted. The diagram shows the key elements of the plasmid, including the CaMV 35 S promoter (for driving gene expression), the *SmSOD* gene (inserted between the BamHI sites), the NOS terminator, and the NeoR/KanR marker for selection. It also depicts the LB T-DNA repeat, RB T-DNA repeat, and restriction enzyme sites (BamHI, HindIII, EcoRI) used for cloning. The total length of the plasmid is 13,414 base pairs.

After successfully isolating the *SmSOD* gene, its sequence was digested from the pTG19 vector using the BamHI enzyme and afterward inserted into the pBI121 expression vector. This pBI121 vector was designed to express the carried gene in plant cells. Similar to the pTG19 vector, cloning results with pBI121 also appeared as white colonies on the plate (Fig. S1b). Subsequently, digestion was performed with HindIII and EcoRI enzymes. According to the vector map pBI121 + *SmSOD* (Fig. 2), the expected fragment lengths are 1680 bp and 11,734 bp (Fig. S2b). Additionally, to further confirm the cloning, the gene carrier vector was digested with the BamHI enzyme, resulting in fragments of 462 bp and 12,952 bp (Fig. S2c).

### Plant inoculation with gene-carrying bacteria and Transgenic plant development

**Fig. 3:**
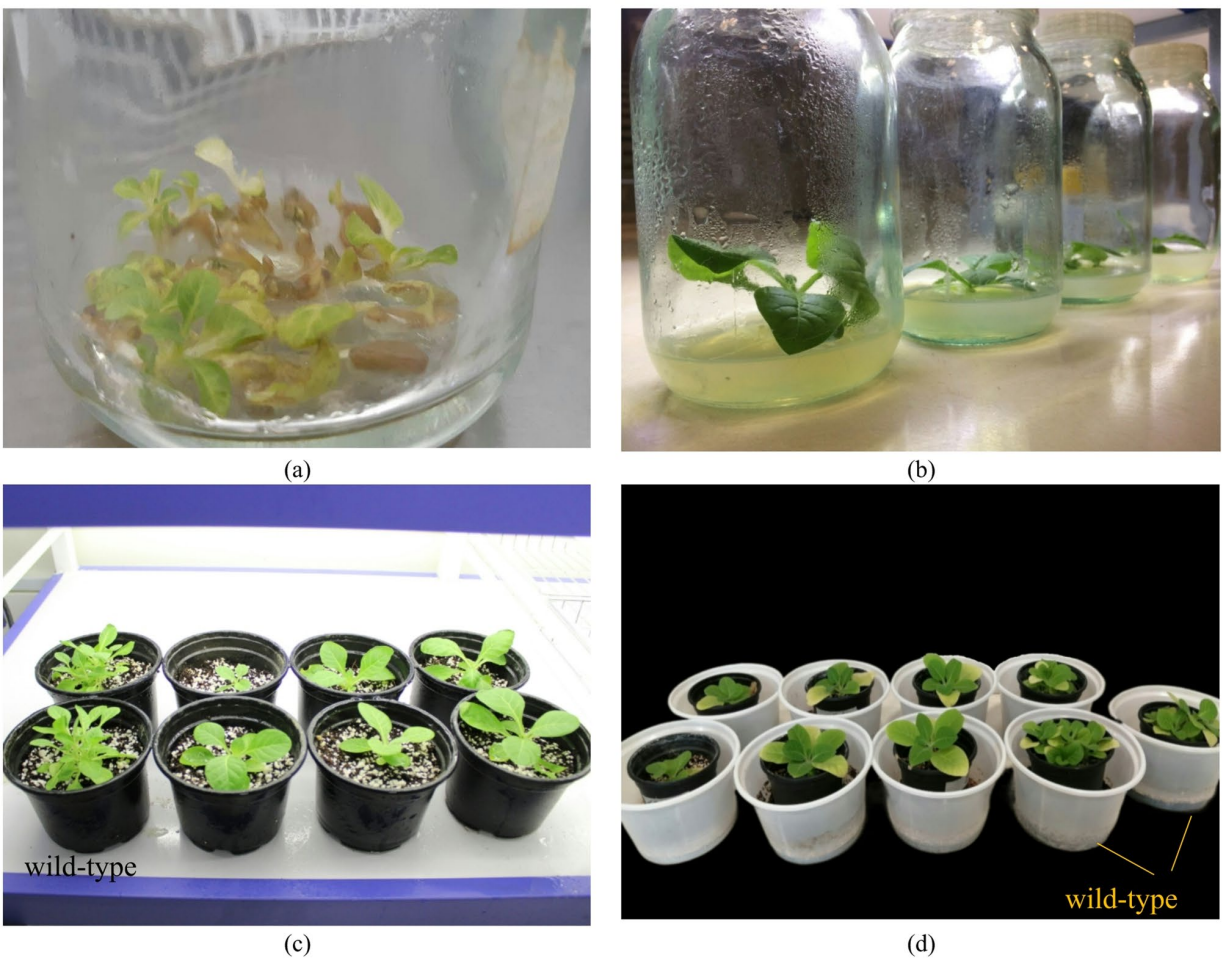
The process of transformation and application of drought stress in tobacco plant. **a** Selection of transgenic plants in selective medium containing antibiotics. **b** Root and shoot induction by hormones in transgenic plants. **c** Adaptation of transgenic plants to soil media in laboratory conditions. **d** Application of drought stress to adapted tobacco seedlings.

The expression vector was introduced into *Agrobacterium tumefaciens* to deliver the *SmSOD* gene into Tobacco plant cells via inoculation. Subsequently, plant tissues were cultured in a selective MS medium containing Kanamycin and Cefotaxime. Tissues lacking the transgene wilted and turned brown, whereas tissues containing the transgene proliferated successfully in the selective medium (Fig. 3a). Wild-type plants were also cultured under the same MS conditions; however, since they did not carry the transgene, no selection with antibiotics was applied. The seedlings developed roots and shoots (Fig. 3b) and were then transferred to pots and a controlled laboratory environment for acclimatization (Fig. 3c). These acclimated seedlings were subsequently subjected to drought stress (Fig. 3d). In Fig. 3, wild-type samples are also indicated, allowing a clear visual comparison with transgenic plants.

### Confirmation of transgenicity of tobacco seedlings by PCR

To confirm the transgenic status of the tobacco seedlings, PCR was performed using two specific primer pairs targeting the *SmSOD* and *NPTII* genes. Prior to using these primers, PCR was performed on wild-type (WT) tobacco plants, and no amplification was observed, confirming that the primers do not amplify endogenous SOD genes. Gel electrophoresis revealed two distinct bands, measuring 462 bp and 350 bp, corresponding to the *SmSOD* and *NPTII* sequences, respectively, thereby confirming the transgenic nature of the analyzed seedlings (Fig. S2c).

### Effect of the *SmSOD* gene on drought stress tolerance in Transgenic plants

#### Gene expression analysis by qRT-PCR

The comparative expression of the *SmSOD* target gene, using 18 S rRNA as a reference, was analyzed on both transgenic and wild-type Tobacco plants exposed to water scarcity stress, and the results were compared with the plants under control conditions. The level of *SmSOD* expression in transgenic plants subjected to drought stress significantly increased (17.012-fold increase) at a 0.01 significance level. Moreover, when evaluating drought stress tolerance, our results demonstrated that the *SmSOD* expression in transgenic plants was almost five times higher than in wild-type counterparts (Table 2).

**Table 2:** Gene expression analysis for *SmSOD* and *18SrRNA* in control and drought stress conditions. Reaction efficiency values represent the efficiency of the PCR reactions, and expression values are normalized to the *18SrRNA* reference gene. The p-value indicates statistical significance, with thresholds of *p* < 0.01. A denotes that the *SmSOD*-T sample group is significantly different from the control group (*p* < 0.01). UP indicates upregulation of *SmSOD* expression under drought stress conditions.

Gene	Type	Reaction efficiency	Expression	*p*-value	Result
*SmSOD* -W	TRG	0.98	3.373	0.240	
*SmSOD* -T	TRG	0.99	17.012^*a*^	0.001	UP
*18SrRNA*-W	REF	0.97	1.639		
*18SrRNA*-T	REF	0.98	1.975		

^a^ SOD-T sample group is significantly different to control group.

#### SOD enzyme activity

**Fig. 4:**
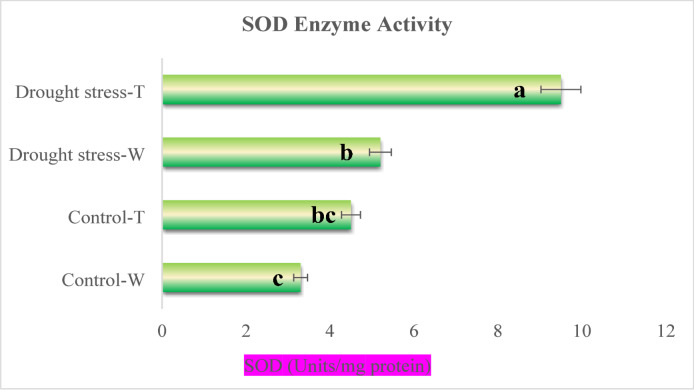
SOD enzyme activity in transgenic tobacco plants under drought stress. Different letters above bars indicate significant differences at *p* < 0.05, as determined by Duncan’s multiple range test. Error bars represent standard error (SE).

The enzymatic activity of *SOD* in transgenic plants exposed to water scarcity conditions was significantly increased (9.5 ± 0.4 Units/mg protein) compared with its activity in the control group (4.5 ± 0.2 Units/mg protein). In wild-type plants, the enzyme activity under drought stress rose to 5.2 ± 0.3 Units/mg protein, compared with 3.3 Units/mg protein in their control group. This represented an 82.6% increase in *SOD* enzyme activity in transgenic plants subjected to water deficiency compared with the wild-type plants. The highest to lowest observed *SOD* enzymatic activities were for drought-subjected transgenic plants, drought-subjected wild-type plants, control transgenic plants, and control wild-type plants, respectively (Fig. 4).

#### Photosynthetic functionality influenced by *SmSOD* transferring to tobacco plants

**Fig. 5:**
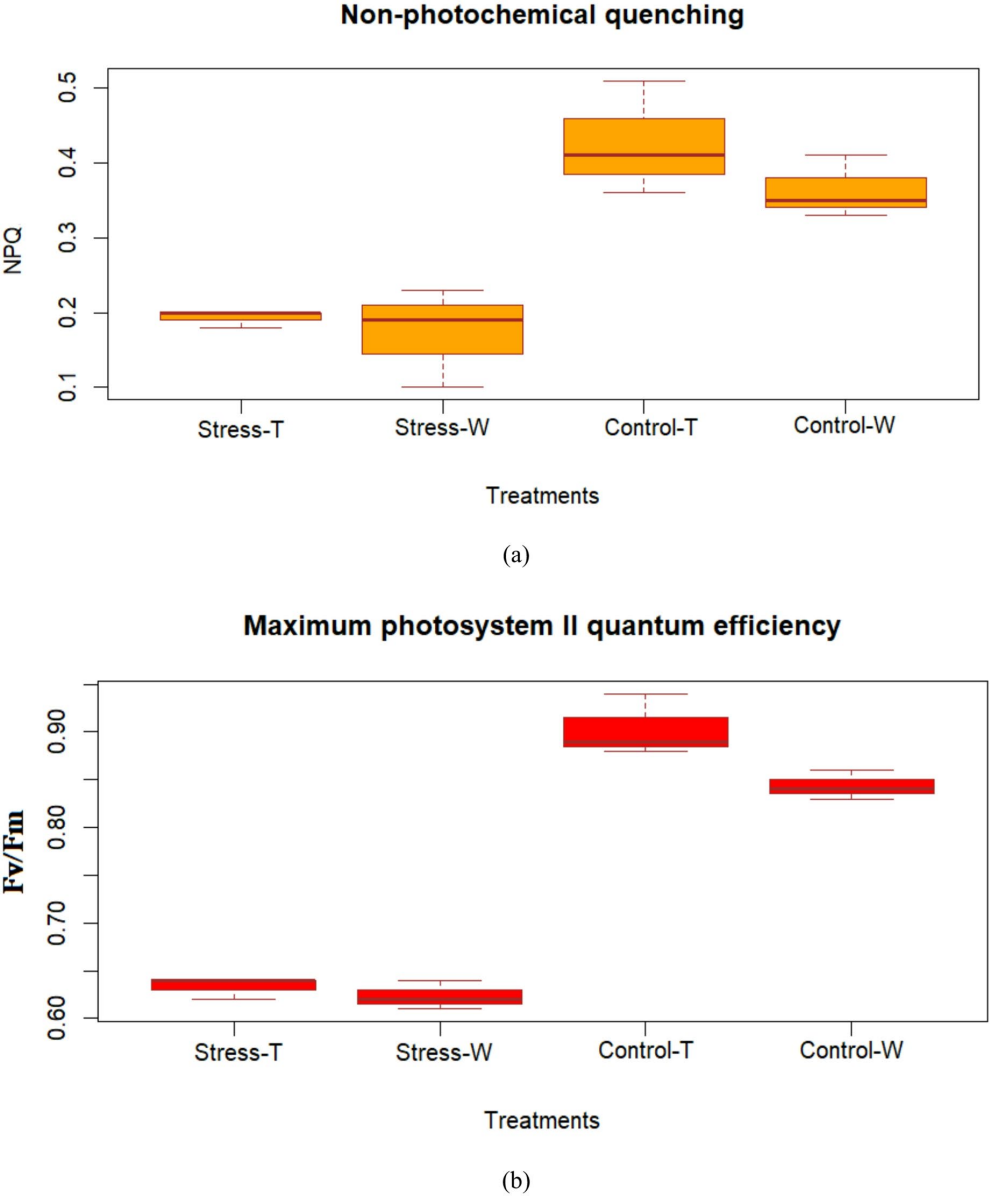
**a** Non-photochemical quenching (NPQ) and **b** Maximum photosystem II quantum efficiency derived from chlorophyll fluorescence parameters exhibited by leaves of tobacco plants in response to drought stress. Statistical significance is indicated at *p* < 0.05, as determined by Duncan’s multiple range test, and error bars represent the standard error (SE).

To assess how the *SmSOD* gene affects the resilience of transgenic plants to stress, an investigation of the plant’s photosynthetic apparatus functionality was performed. This study focused on two critical features related to the F_v_/F_m_ and the protective stress mechanism: Non-photochemical quenching light steady-state (NPQ.lss) and Maximum Quantum Yield (QY_max_). A significant increase (*p* < 0.05) in NPQ levels was observed in both transgenic and wild-type plants exposed to water scarcity conditions compared with their levels in the control group (Fig. 5a). The average NPQ in transgenic plants under water deficiency conditions was 0.426 ± 0.04, while in non-stressed transgenic plants, it was 0.193 ± 0.01. In wild-type plants, the average NPQ under water scarcity conditions was 0.363 ± 0.03 and 0.173 ± 0.01 in non-stressed conditions. The percentage increase in the NPQ under drought stress was 120.72% for transgenic plants and 109.82% for wild-type plants. Additionally, transgenic plants exposed to water scarcity stress showed 17.3% higher NPQ than wild-type plants. Transgenic plants had 11.5% higher NPQ in control conditions than the wild-type group.

Parallel to the NPQ observations, a significant increase (*p* < 0.05) in QY_max_ (also known as the value of F_v_/F_m_) values was observed in both transgenic and wild-type plants under water deficiency conditions compared with controls. The difference in F_v_/F_m_ QY_max_ values under water scarcity conditions compared with control conditions was recorded at 0.27 ± 0.01 for transgenic plants and 0.22 ± 0.01 for wild-type plants. The increase in QY_max_ attributable to drought stress was calculated at 42.65% for transgenic plants and 35.31% for wild-type plants. In a comparative analysis under water scarcity conditions, transgenic plants had a 7.11% higher F_v_/F_m_ QY_max_ than wild-type plants. Additionally, there was a slight increase of 1.60% in F_v_/F_m_ QY_max_ in control group plants (Fig. 5b).

**Fig. 6:**
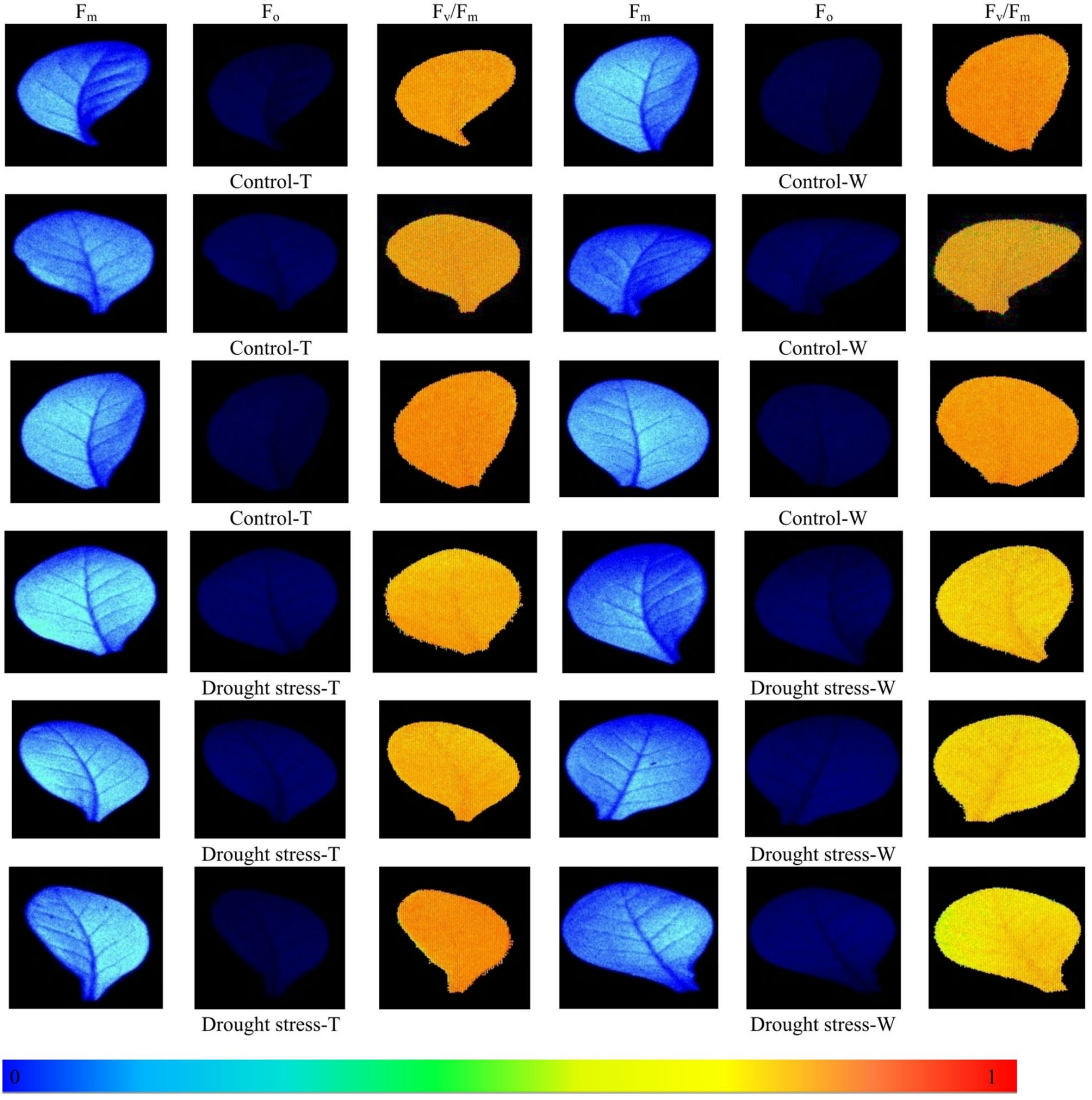
Representative images of maximum quantum yield of photosystem II (F_v_/F_m_) obtained from the leaves of tobacco plants in response to drought stress. The color scale in the bottom of the image represents the level of F_v_/F_m_. Close to warmer color (close to 1) indicates higher F_v_/F_m_ and close to cooler colors indicates lower F_v_/F_m_.

F_v_/F_m_ was significantly compromised in wild-type plants under water scarcity stress. In contrast, transgenic plants exposed to the same conditions showed a lesser degree of reduction (Fig. 6). The F_v_/F_m_ ratio in wild-type plants showed a significantly greater decrease under drought stress compared with the F_v_/F_m_ ratio in transgenic plants exposed to the same conditions. In the evaluation of F_v_/F_m_ imaging, plants subjected to drought stress typically showed a higher F_v_/F_m_ (brighter yellow hue), in contrast to control plants, which present a redder coloration (Based on the scale provided close to warmer color indicate higher F_v_/F_m_ and close to cooler color indicate lower F_v_/F_m_.

#### Stomatal morphology influenced by *SmSOD* transformation

**Fig. 7:**
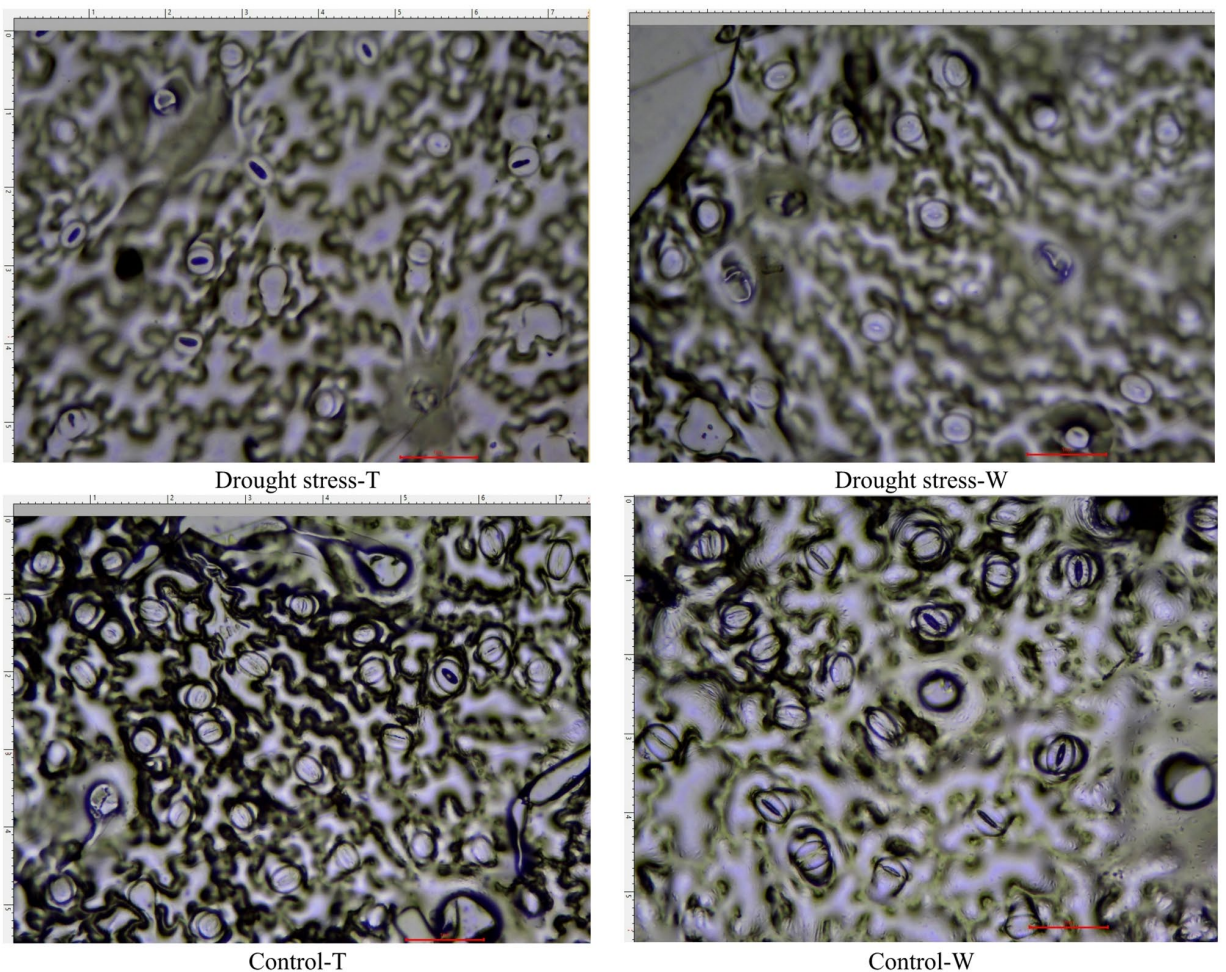
Stomatal anatomy on transgenic tobacco leaves from plants grown under drought stress. The scale bar unit is in nanometers (um). SEM images at 10,000X magnification.

**Fig. 8:**
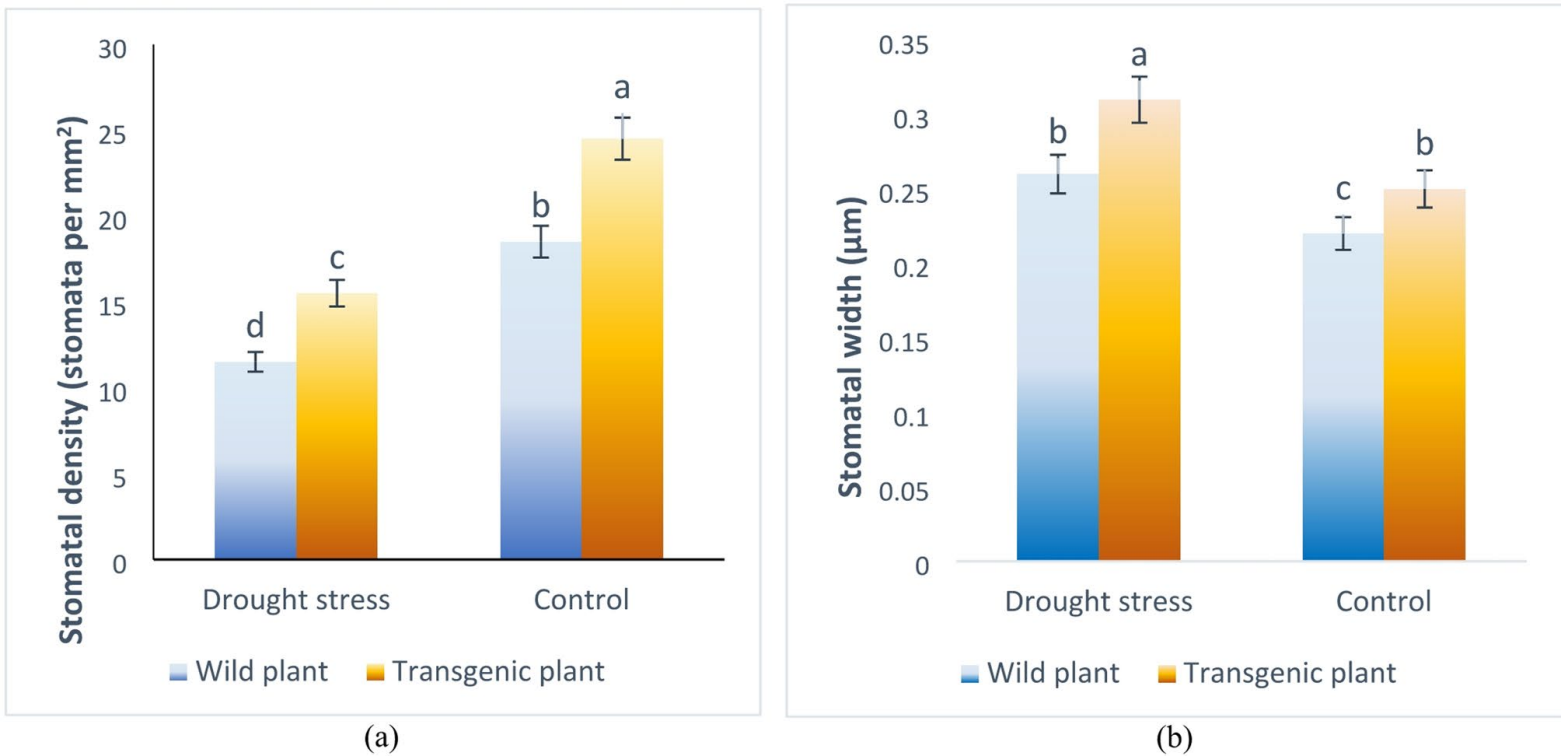
Stomatal characteristics of wild-type and transgenic tobacco plants under control and drought stress conditions. **a** Stomatal density (stomata per mm^2^). **b** Stomatal width (µm). Different letters above bars indicate significant differences at *p* < 0.05, as determined by Duncan’s multiple range test. Error bars represent standard error (SE).

The images prepared for apertures in four samples (Drought stress-wild plants, Drought stress-transgenic plants, Control- Wild plants, Control- Transgenic plants) are presented in Fig. 7. Observations revealed that plants subjected to drought stress had a lower stomatal density than those grown under control conditions (Fig. 8a). The stomatal density measured for transgenic and wild-type plants was 24.5 and 18.5 stomata per mm^2^, respectively, under control conditions. However, under drought stress, the stomatal density in transgenic and wild-type plants decreased to 15.5 and 11.5 stomata per mm^2^, respectively.

The percentage decrease in stomatal density was 58% for transgenic plants and 60.8% for wild plants. In contrast to the pattern observed in stomatal density, the width of stomata in both transgenic and wild-type plants increased under drought conditions. In transgenic plants, the stomatal width expanded from 0.31 ± 0.02 μm to 0.25 ± 0.01 μm under drought stress, while in wild-type plants, it decreased from 0.26 ± 0.01 μm to 0.22 ± 0.01 μm. The decrease in stomatal width under drought stress was 24% for transgenic plants and 18.1% for wild-type plants. Notably, under drought conditions, the stomatal width of transgenic plants was 19.2% smaller than that of wild-type plants (Fig. 8b). Furthermore, the analysis of stomatal length in both transgenic and wild-type plants subjected to either drought or control conditions revealed no significant differences, with measurements ranging between 0.3 ± 0.02 μm and 0.5 ± 0.03 μm.

## Discussion

This study compared transgenic and wild-type plants subjected to drought stress and under control conditions. Three key parameters, including *SmSOD* gene expression, SOD enzyme activity, plant photosynthetic system functionality, and stomatal morphology in response to drought stress, will be discussed.

### *SmSOD* gene expression and enzyme activity

*SOD* catalyzes the conversion of superoxide into hydrogen peroxide and oxygen, making it an essential component of the plant antioxidant defense system^66^. The results of this study showed that in transgenic plants under drought stress (*SmSOD* -T), *SmSOD* expression was significantly upregulated, reaching 17.012-fold relative to the control (*P* = 0.001). In contrast, wild-type plants under drought stress (*SmSOD* -W) showed no significant change (3.373-fold, *P* = 0.240). Moreover, the comparison between transgenic and wild-type plants under drought stress revealed that *SmSOD* expression in transgenic plants was nearly five times higher than in wild-type plants. These findings confirm the successful expression of the transferred gene and its role in enhancing drought tolerance. This result aligned with our expectations and confirmed the successful expression of the transferred gene. Other studies have documented similar results that have expressed this gene in different plants. Lin et al.^67^ reported that overexpressing *SOD* genes derived from *Arabidopsis* and winter squash improves chilling tolerance through ABA-sensitive transcriptional mechanisms in transgenic *Arabidopsis*. Findings indicated that *Arabidopsis* seedlings with elevated levels of *AtSOD* and *CmSOD* showed significantly higher tolerance to chilling stress and reduced oxidative damage compared with wild-type plants subjected to cold conditions. This evidence suggests that overexpressing *AtSOD* and *CmSOD* in *Arabidopsis* may facilitate chilling tolerance by effectively removing superoxide radicals (O_2_^·−^). Drought-induced ABA accumulation has been shown to activate antioxidant enzymes, including SOD, through ABA-dependent signaling cascades^68^. Thus, the upregulation of *SmSOD* in transgenic plants may function in coordination with ABA-mediated regulatory pathways to optimize stomatal behavior, sustain photosynthetic efficiency, and maintain redox homeostasis under drought conditions.

Gupta et al.^69^ showed that overexpressing pea-derived *SOD* in tobacco plants provided better protection against oxidative stress. The results revealed that *SOD* + plants maintained higher photosynthesis rates compared to *SOD*- plants at all growth stages, with *SOD* activity nearly three times higher in *SOD* + plants. Additionally, leaf discs from SOD- plants, which did not express pea *SOD*, exhibited significantly reduced photosynthesis. Various studies have emphasized the importance of *SOD* in conferring tolerance to abiotic stresses^70,71^. In this study, the highest enzymatic activity was observed in transgenic plants under drought conditions, 1.8 times higher than in wild-type plants.

Elevated SOD activity in the chloroplasts of transgenic tobacco plants provides better protection against membrane damage induced by methyl viologen (MV). Additionally, overexpression of Cu/Zn *SOD* improves plant resilience against photooxidative damage under intense light and low temperature conditions^72^. Transgenic plants co-expressing *SOD* and *APX* in chloroplasts exhibit greater tolerance to oxidative stress, with higher activities of both *APX* and *SOD* compared to wild-type plants^73^. Other studies have also reported increased SOD activity in transgenic plants under abiotic stress^74,75^.

### Photosynthetic functionality and photoprotective mechanisms enhanced by *SmSOD* transformation in tobacco plants

The photosynthetic functionality of the plant was examined. The parameters investigated in this study included photosynthetic functionality (F_v_/F_m_), photoprotective mechanisms (NPQ), and stomatal characteristics (morphology, density, and dimensions). Regarding photosynthetic functionality, transgenic plants had higher values for F_v_/F_m_ than wild-type plants. The higher Fv/Fm values observed in *SmSOD*-overexpressing tobacco plants indicate improved photosystem II (PSII) efficiency and reduced photoinhibition under drought stress. This enhancement suggests that *SmSOD* overexpression contributes to maintaining chloroplast integrity and protecting the PSII reaction centers from oxidative damage through efficient ROS scavenging. Van Beek et al.^76^ investigated the impact of overexpressing the *SlNAC2* transcription factor on the photosynthetic apparatus of tobacco plants under water scarcity conditions. The results showed that both stomatal conductance and Fv/Fm were reduced in transgenic and wild-type plants under water stress, while these parameters remained stable in well-watered control plants. Jia et al.^77^ also demonstrated that Fv/Fm decreased under drought stress. In this study, Fv/Fm values declined in plants under stress treatments. Some studies reported a significant increase in Fv/Fm due to water scarcity^78^, while others found no significant changes^79^.

Regarding photoprotective mechanisms, higher NPQ levels were identified in transgenic plants exposed to water scarcity than in wild-type plants. NPQ refers to the fraction of light energy captured through antenna pigments that are not employed for electron transfer and dissipated as heat, mainly through carotenoids. Higher NPQ values are crucial for dissipating surplus energy^80^, which lets photosystems eliminate the extra energy input. The increased NPQ values in transgenic plants reflect a strengthened photoprotective mechanism, which facilitates the dissipation of excess excitation energy as heat, thereby preventing ROS overproduction. These physiological responses are closely linked to the antioxidant defense system, where SOD acts as the first enzymatic barrier converting superoxide radicals into less harmful molecules. Glowacka et al.^81^ showed that lowering excitation pressure in PSII through enhanced NPQ can effectively reduce excitation pressure under light conditions. In tobacco, NPQ enhancement was achieved by constitutively overexpressing *PsbS* in PSII^82^. Liu et al.^83^ explored the synergistic effects of cold and drought stress on photosynthetic activity and osmotic regulation in *Elymus nutans* Griseb, finding significant decreases in PSII electron transport rate and NPQ, indicating reduced energy dissipation and increased vulnerability of the photosystem. These results aligned with other studies showing no significant changes in Fv/Fm or NPQ under drought conditions^84^. Similar findings were reported in potatoes, chickpeas, tobacco, maize under drought, and soybeans exposed to cold stress^85,86^. Conversely, Gallie and Chen^87^ found that mutations in *FSD2* led to elevated superoxide production, decreased chlorophyll content, and reduced PSII efficiency in *Arabidopsis*.

In examining stomatal characteristics, both transgenic and wild-type plants showed significantly lower stomatal density under water scarcity compared to control plants, while stomatal width was greater in stressed plants, with transgenic plants having wider stomata than wild-type plants. Water loss through stomatal transpiration is essential for regulating leaf temperature and indicates the plants’ ability to retain water and tolerate drought conditions^88^. Transgenic plants had higher stomatal conductance under drought stress compared to wild-type plants, and as stress intensified, significant changes were observed in stomatal length, width, and density.

Differing stomatal ROS responses influence stomatal closure in barley, revealing various drought regulation strategies^89^. Stomatal closure is one of the initial responses to water scarcity, significantly limiting photosynthesis^90^. Drought-tolerant genotypes exhibited slower wilting rates and increased water use efficiency (WUE), due to reduced water loss from stomatal closure, which helps maintain soil moisture. Under normal conditions, these genotypes showed higher stomatal width and greater density compared to drought-sensitive genotypes^91^.

Vulnerable wild barley genotypes showed higher stomatal density in response to drought stress, but a decrease in stomatal dimensions was observed^92^. In contrast, grapevine cultivars exhibited higher stomatal density and smaller stomata under drought conditions^93^. Zhang et al.^94^ showed that overexpression of *TaMnSOD* in transgenic cotton increased drought tolerance, leading to higher transpiration, stomatal conductance, and net photosynthesis. Similarly, Pal et al.^95^ demonstrated that overexpression of *PaSOD* in transgenic potatoes enhanced photosynthetic efficiency under drought stress. These results align with findings in tobacco, where successful transfer and expression of the *SmSOD* gene improved photosynthetic efficiency in transgenic plants.

## Conclusion

Water scarcity and the increasing prevalence of drought have a profound impact on the environment, significantly affecting agriculture and food security. Addressing this issue is crucial to prevent further deterioration of global food supply systems. Biotechnological advancements, particularly in genetic engineering, offer promising approaches to mitigate these challenges. In this study, a key gene associated with tolerance to both biotic and abiotic stresses was transferred from a resistant plant species (Milk thistle) to a sensitive species (Tobacco). The *SmSOD* gene utilized in this research belongs to the *Cu/ZnSOD* type, which, compared to other SOD isoforms such as *MnSOD* and *FeSOD*, plays a broader role in regulating oxidative balance within various organelles, including chloroplasts, the cytosol, and peroxisomes. The use of the *SmSOD* gene as a *Cu/ZnSOD* represents a significant novelty in this study, as no prior research has investigated this specific isoform from *Silybum marianum* or evaluated its function in interspecies gene transfer. The objective of this research was to assess the effectiveness of this genetic modification in enhancing drought stress tolerance. The results demonstrated that *SmSOD* gene transfer positively influenced photosynthetic performance and photoprotective mechanisms in tobacco plants, thereby improving their stress resilience. Based on these findings, applying this gene transfer strategy to other sensitive crop species is recommended. Incorporating stress-tolerant genes from resistant plants could potentially enhance crop resilience to environmental stresses, thereby promoting sustainable agriculture and contributing to global food security.

## Supplementary Information

Below is the link to the electronic supplementary material.

Supplementary Material 1

## Data Availability

The nucleotide sequence of the *SOD* gene generated during the current study is publicly available in the NCBI GenBank repository under the accession number MG893090.
