# Supplementary material for: Overexpression of milk thistle SOD gene enhances drought tolerance in tobacco by improving photosynthesis and photoprotection
Source: Sci Rep. 2025 Dec 11;16:1833. doi: 10.1038/s41598-025-31510-3 (PMC12804998; doi:10.1038/s41598-025-31510-3)
Supplement: Supplementary file 1 — Supplementary Material 1 [file 41598_2025_31510_MOESM1_ESM.pdf]

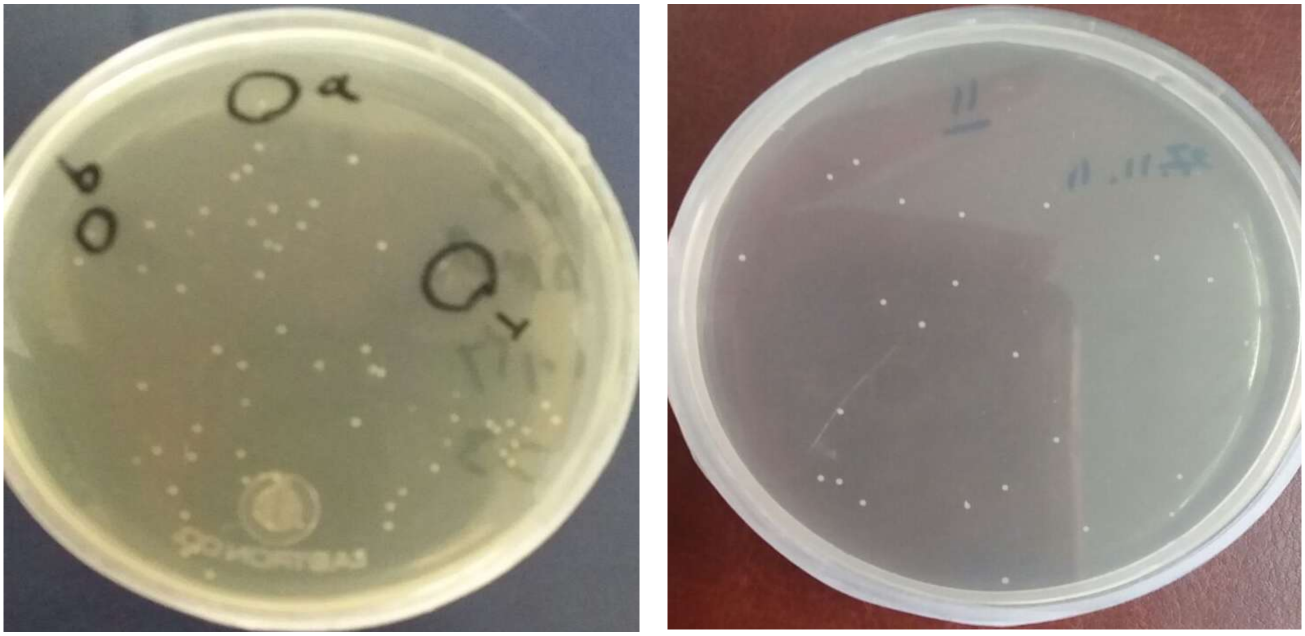

(a) (b)

Figure S1. Cloning confirmation of the SOD gene into the vectors pTG19 and pBI121 transformed in E. coli DH5α bacteria through the growth of bacterial colonies containing the desired vector. (a) Bacterial colonies containing vector pTG19+SOD. (b) Bacterial colonies containing vector pBI121+SOD.

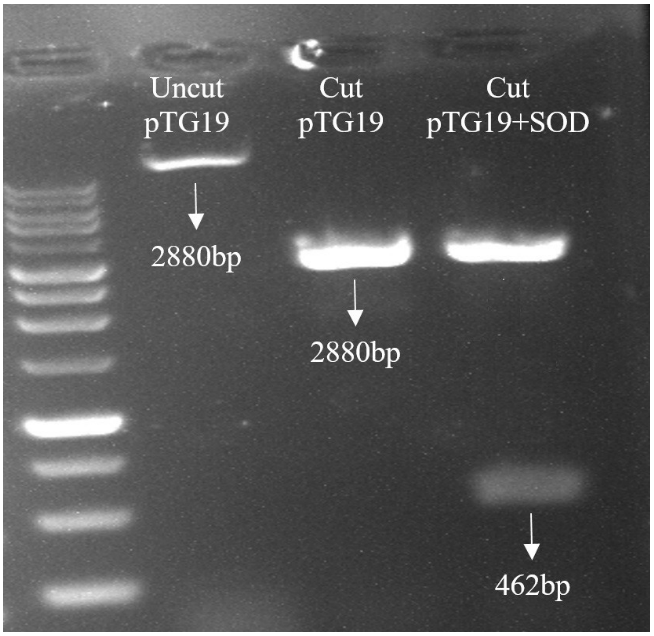

(a)

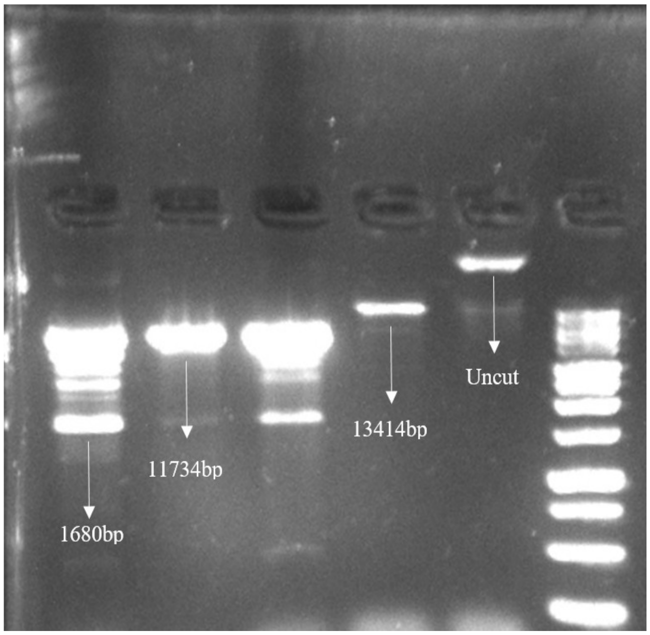

(b)

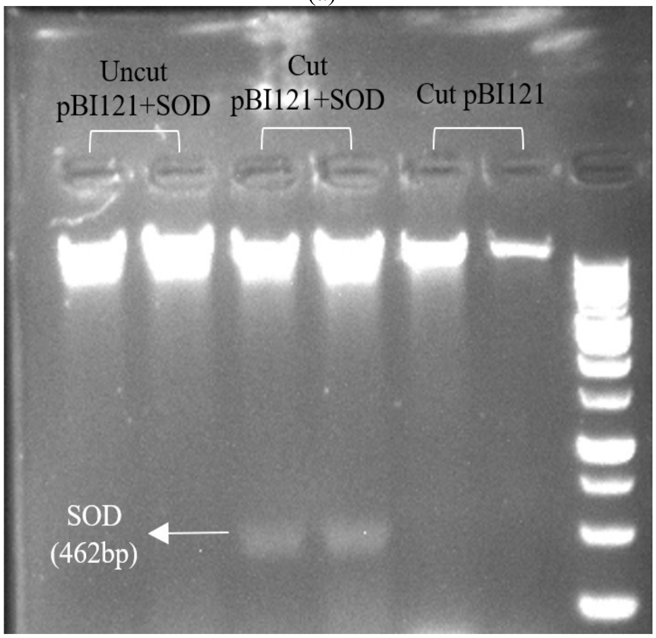

(c)

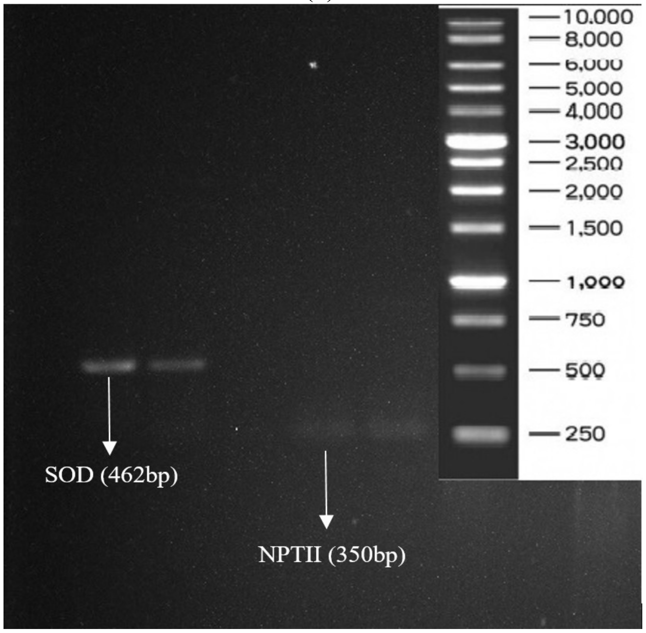

(d)

Figure S2. Confirmation of vectors digestion pTG19 and pBI121 and PCR results on gel electrophoresis. (a) Digestion of pTG19+SOD vector with BamHI enzyme. (b) Digestion of pBI121+SOD vector with HindIII and EcoRI enzymes. (c) Digestion of pBI121+SOD vector with BamHI enzyme. (d) Confirmation of PCR results of transgenic plants with primers SOD and NPTII.
